# Biosorption Potential of *Phanerochaete chrysosporium* for Arsenic, Cadmium, and Chromium Removal from Aqueous Solutions

**DOI:** 10.1002/gch2.201800064

**Published:** 2018-10-25

**Authors:** Darshan M. Rudakiya, Vignesh Iyer, Darsh Shah, Akshaya Gupte, Kaushik Nath

**Affiliations:** ^1^ Department of Microbiology N. V. Patel College of Pure & Applied Sciences Vallabh Vidyanagar 388 120 Anand Gujarat India; ^2^ Department of Chemical Engineering G. H. Patel College of Engineering & Technology Vallabh Vidyanagar 388 120 Anand Gujarat India

**Keywords:** biosorption, heavy metals, *Phanerochaete chrysosporium*, response surface methodology

## Abstract

Efficient degradation of hazardous contaminants from contaminated water is the major challenge for researchers, wherein heavy metals are the prominent contaminants. Consequently, the assessment of multimetal removal is necessary using efficient biosorbant. In this work, the capability of *Phanerochaete chrysosporium* is evaluated for the individual and simultaneous removal of heavy metals. Individual and simultaneous removal of As, Cd, and Cr is optimized using response surface methodology based on the central composite design by changing the variables, i.e., pH, fungal biomass, and metal concentration. Optimization of the individual metal removal study reveals that fungus effectively absorbs As (29.95 mg L^−1^), Cd (18.1 mg L^−1^), and Cr (26.34 mg L^−1^) at 6.1, 5.64, and 4.15 of pH, respectively. Similarly, As (14.18 mg L^−1^), Cd (4.53 mg L^−1^), and Cr (9.28 mg L^−1^) are absorbed by fungal hyphae simultaneously within 1 h. Changes in the morphology of fungal hyphae are detected in metal absorbed samples as compared to the control hyphae. Interaction of metal‐absorbed fungal hyphae is analyzed using FTIR spectroscopy, revealing that the proteins, carbohydrates, and fatty acids present in the fungal cell are interacted with metals. The model white rot fungi used in the present study can be applied efficiently for the multimetal removal in effluent treatment plants.

## 1 Introduction

In the last decade, the presence of heavy metals in wastewater generated from a number of chemicals and metallurgical units has posed a serious environmental and occupational concern.^1^ Barring a selected few as beneficial trace elements, majority of them have no established biological functions and are considered as nonessential metals. Because of their high degree of toxicity, lead (Pb), arsenic (As), cadmium (Cd), chromium (Cr), selenium (Se), and nickel (Ni) rank among the priority metals that are of great public health significance.^2^ These are all systemic toxicants inducing multiple organ damage, even at lower levels of exposure. According to the United States Environmental Protection Agency and the International Agency for Research on Cancer, these metals are also classified as either “known” or “probable” human carcinogens based on epidemiological and experimental studies, depicting an association between exposure and cancer incidence in humans and animals.^3^ Heavy metal exposure, in particular, affects all organ systems including the nervous, dermatologic, cardiovascular, gastrointestinal, and respiratory systems.^3^, ^4^, ^5^

Mostly, the waters of highly metal‐contaminated sites and abandoned mines are in acidic range due to the higher concentration of sulphates, metals, and metalloids. White rot fungal communities may play crucial role in removal of heavy metals as they grow in acidic medium and survive with higher concentration of heavy metals.^6^ Additionally, white rot fungi accumulate organic acids, carboxylic, and thiol ligands and other polymeric substances extracellularly, which reduce the toxicity of heavy metals.^7^, ^8^ *Phanerochaete chrysosporium*, a representative white rot fungus, has been used extensively for environmental engineering fields as its favorable metal absorption ability.^8^, ^9^, ^10^ In the present study, free cells of *P. chrysosporium* are used for metal absorption, which offers several advantages over living cell study: i) wide range of operating conditions (pH and temperature), ii) no nutrition requirement, iii) comparative fast metal removal (in terms of time), and iv) resistant to initial higher metal concentration.^6^

In biosorption studies, biosorbent concentration, pH, temperature, and metal concentration are the most important parameters, which directly affect the biosorption efficacy of metals. Previously, various researchers studied the biosorption efficiency of fungal biomass, optimized using either OFAT (one factor at a time) method or multivariate optimization methods, wherein multivariate optimization have more advantages as compared to conventional methods. In multivariate optimization, response surface methodology (RSM) leads to an empirical mathematical model that narrates the metal removal with different parameters and their interactions.^11^, ^12^ Several studies depicted the multimetal removal efficiency using multivariate optimization approach.^12^, ^13^, ^14^ However, some of them used fungal biomass for the multimetal removal and little is known about the metal–fungal interactions during biosorption.^15^ Thus, the impetus for the present work was stimulated first by the biosorption potential of *P. chrysosporium* for efficient removal of As^3+^, Cd^2+^, and Cr^6+^, and second to obtain a further insight into the sorption properties of biosorbent.

### 2 Results and Discussion

### 2.1 Metal Removal by *P. Chrysosporium*

**Figure 1:**
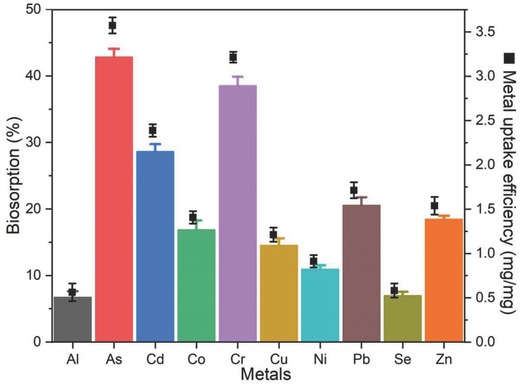
Biosorption (%) and metal removal efficiency (mg of metal mg^−1^ of fungal biomass; black square) of *P. chrysosporium* hyphae within 1 h at 30 °C and 120 rpm.

As described earlier, pH, temperature, metal, and biomass concentration, agitation and contact time are the most important parameters for the metal biosorption study.^11^, ^13^ *P. chrysosporium* showed highest biosorption toward metals at 30 °C and 120 rpm (Figure S1, Supporting Information), wherein As and Cr were used for the biosorption study. Higher temperature and agitation may distort the fungal hyphae, which affect the efficacy of biosorption.^12^ Based on the primary screening of different heavy metals, white rot basidiomycete *P. chrysosporium* exhibited the higher absorption capability toward As (21.43 mg L^−1^), Cr (19.28 mg L^−1^), Cd (14.32 mg L^−1^), and Pb (10.28 mg L^−1^) within 1 h at 30 °C and 120 rpm. As shown in **Figure** **1**, metal uptake efficiency of the fungi was higher in case of As (3.57 mg mg^−1^), Cr (3.21 mg mg^−1^), Cd (2.38 mg mg^−1^), and Pb (1.71 mg mg^−1^). Hyphal cells of *P. chrysosporium* could easily absorb the metal ions under acidic condition; hence, fungi are known to grown in the acidic environment.[[qv: 6a]] Fungal cells can remove the metal ions from the aqueous solutions either by binding with the components of cell wall or by utilizing the metal ions for the cell metabolism. Similar studies have been carried out to check the metal removal efficacy of *P. chrysosporium*, wherein fungus exhibited higher removing capacity toward Pb, Cd, Cu, Ni, and Hg.^17^, ^18^, ^19^

### 2.2 Response Surface Analysis

The experimental design of biosorption performed by RSM method enables us to investigate the interaction between pH, metal concentration, and fungal biomass, which had great influence on metal biosorption. The data of individual and simultaneous metal biosorption were analyzed by a quadratic multiple regression using Design‐Expert v10.0.5. For all biosorption studies (individual and simultaneous), the statistical significance of quadratic model for the biosorption responses was calculated using the analysis of variance (ANOVA). The results of ANOVA and *R* ^2^ values for As, Cd, and Cr biosorption studies (individual and simultaneous) are shown in Tables S2 and S3 in the Supporting Information. ANOVA results study suggested that polynomial quadratic model of all metal biosorption studies was significant with *p*‐value of <0.0001. In addition, *R* ^2^ values for all polynomial quadratic models were more than 0.96 and the difference is less than 0.2 between predicted *R* ^2^ and adjusted *R* ^2^ values. Furthermore, the lack of fit was insignificant for all models, indicating the excellent correlation between experimental and predicted values of biosorption. The signal to noise ratio is measured by the adequate precision, which is more than 4.0 is desirable.^20^ In all polynomial quadratic models have more than 4.0 ratios, suggesting these models can be used to navigate the design space. Based on the ANOVA table of individual and simultaneous biosorption of As, Cd, and Cr, the predicted response was calculated using the equation that is given in Table S4 in the Supporting Information.

#### 2.2.1 Individual Metal Biosorption Optimization

**Figure 2:**
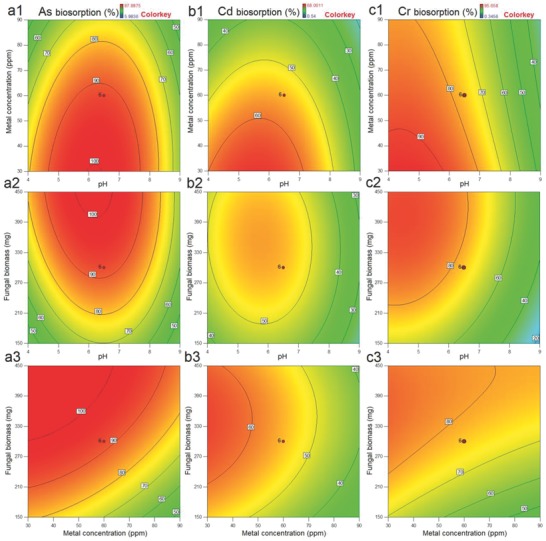
Contour plots for the individual metal removal by *P. chrysosporium* as function of a1, b1, and c1) metal concentration and pH; a2, b2, and c2) fungal biomass and pH; and a3, b3, and c3) fungal biomass and metal concentration.

Contour plots demonstrate the effect of two variables at their studied concentration range with fixed concentration of third variable. In case of As, *P. chrysosporium* exhibited higher biosorption with lower metal concentration and higher concentration of fungal biomass (**Figure** **2**a1–a3). Fungus showed the ≥90% of biosorption efficiency in the wide pH range, including acidic to neutral region of pH (4.7–7.7). The pH of solution has great impact on the biosorption as it influences the charge on the surface of biosorbent.^12^ Overall, fungus could easily remove 30 mg L^−1^ of As within 1 h of incubation. Similarly, fungus had higher Cd biosorption efficiency with higher concentration of biomass and lower concentration of metal (Figure 2b1–b3). However, highest efficiency was 60%, i.e., fungus had capability to remove 18.1 mg L^−1^ of Cd within 1 h under optimized condition. Although the biosorption was lower compared to As, Cd biosorption exhibited in wide range of pH (4.0–7.2) with highest biosorption at 5.94. Higher fungal biomass and lower metal concentration tend to higher biosorption of Cr (Figure 2c1–c3). At low pH, biosorption of As and Cd was found to be low due to the binding of H^+^ ions with fungal hyphae. At higher pH, As and Cr ions compete with OH^−^ ions for the active site of surface.^21^, ^22^ These results demonstrate the applicability of fungus for As and Cd removal in acidic/neutral pH effluents. *P. chrysosporium* showed the higher Cr biosorption compared to Cd; however, pH was ranging from 3.5 to 5.8. It is observed that Cr biosorption was increased with decrease of pH from 6.0 to 4.0. This may be attributed due to electrostatic interactions between fungal hyphae and Cr. At lower pH values, negatively charged surface of fungal hyphae is decreased that may help in binding of Cr to fungal hyphae.^21^, ^23^ Thus, metal biosorption may not proceed through single mechanism as fungi have a complex structured cell wall, which can be differed for different metals.^24^, ^25^, ^26^

#### 2.2.2 Simultaneous Metal Biosorption Optimization

**Figure 3:**
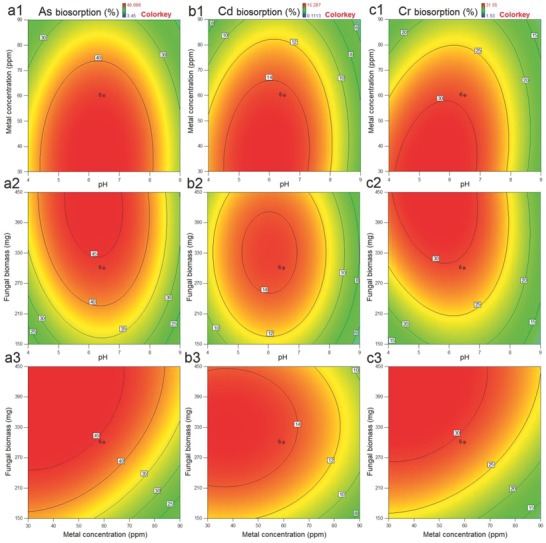
Contour plots for the simultaneous metal removal by *P. chrysosporium* as function of a1, b1, and c1) metal concentration and pH; a2, b2, and c2) fungal biomass and pH; and a3, b3, and c3) fungal biomass and metal concentration.

Responses of simultaneous metal biosorption experiments displayed the different pattern of metal absorption than individual metal biosorption experiments. *P. chrysosporium* exhibited the higher absorption of metals in wide range of pH (4.2–7.8). This is due to the presence of more than one metal in the aqueous solution.^25^ However, the lower metal biosorption is observed than individual metal biosorption experiments (**Figure** **3**a–c). Similar to the preceding experiments, higher concentration of biomass and lower concentration of metal tend to the higher biosorption efficacy. Simultaneously, metal removal efficiency of *P. chrysosporium* for As, Cd, and Cr was 14.15, 4.53, and 9.28 mg L^−1^ within 1 h, respectively.

### 2.3 Validation of the Experiments

**Figure 4:**
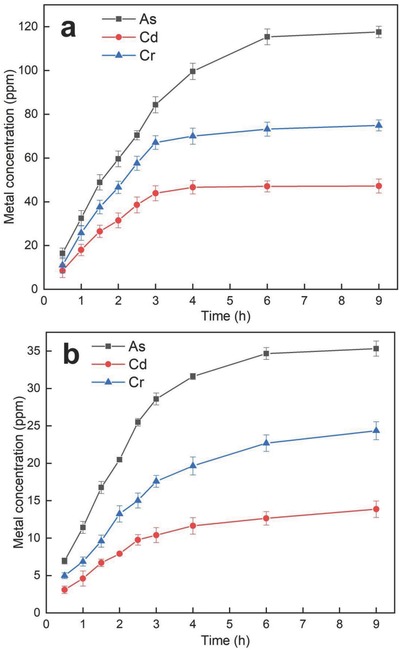
Time course study of metal removal by *P. chrysosporium* under respective optimized conditions for a) individual metals and b) mixed metal solution.

From the optimization data, it is clear that pH of the solution has higher influence on the biosorption efficacy. *P. chrysosporium* exhibited highest individual biosorption of As, Cd, and Cr at 6.16, 5.64, and 4.15, respectively. Thus, we used Na‐phosphate buffer (pH 6.16, 50 × 10^−3^ m) and Na‐acetate buffer (pH 5.64 and 4.15, 50 × 10^−3^ m) for the validation of biosorption experiments. Time course study of individual metal biosorption under respective optimized conditions is shown in **Figure** **4**a. *P. chrysosporium* had efficacy to remove As (117.6 ± 2.6 mg L^−1^), Cd (47.21 ± 2.1 mg L^−1^), and Cr (74.89 ± 2.5 mg L^−1^) from the aqueous solution after 9 h of incubation, showing the higher concentration of fungus. Higher concentration of metal absorption led to the biosorption saturation observed after 3 h of incubation with an exception of As. *P. chrysosporium* showed higher absorption of As even after 3 h of incubation, confirming that As can be utilized by cell metabolism.

In simultaneous removal study, the pH was shifted for all metal biosorption, wherein As, Cd, and Cr were efficiently removed at 6.24, 5.91, and 5.50, respectively. In contrast, low biosorption efficiency was observed as compared to individual metal sorption experiments. Thus, we used the Na‐acetate buffer (pH 5.88, 50 × 10^−3^ m) that is mean of the pH obtained from RSM‐ central composite design (CCD) experiments. Time course study of simultaneous removal of As, Cd, and Cr is shown in Figure 4b, wherein *P. chrysosporium* showed the lower efficacy to remove all metals compared to individual metal biosorption experiments. However, fungus showed the higher As removal efficacy (35.33 ± 1.3 mg L^−1^), moderate Cr removal efficacy (24.35 ± 1.5 mg L^−1^), and lower Cd removal efficacy (13.87 ± 1.2 mg L^−1^) after 9 h of incubation. *P. chrysosporium* exhibited the absorption saturation of As, Cd, and Cr after 4 h of incubation, suggesting the multimetals may bind with the components of cell wall and may block the functionally active sites; subsequently other metal ions may not bind with the same site.

### 2.4 Scanning Electron Microscopy (SEM) and Energy‐Dispersive X‐Ray Analysis (EDXA) Analysis

**Figure 5:**
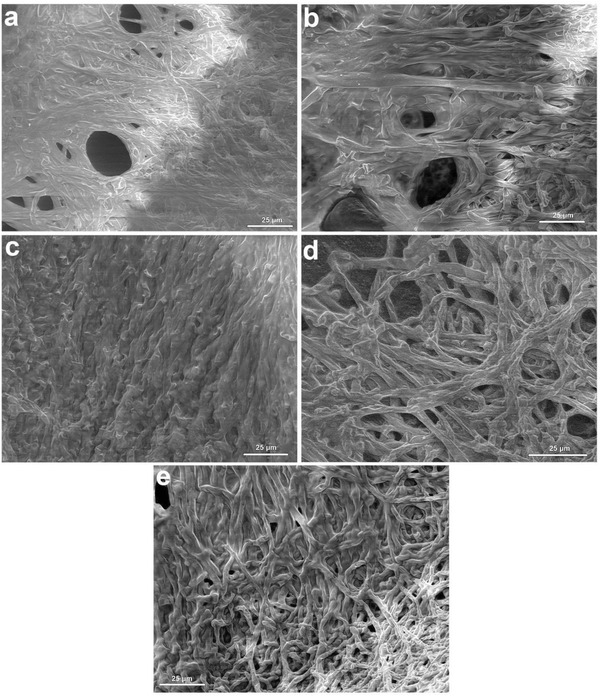
Scanning electron microscopic image of a) control fungal hyphae, b) As absorbed hyphae, c) Cd absorbed hyphae, d) Cr absorbed hyphae, and e) mixed metal absorbed hyphae.

Scanning electron microscopic images of control *P. chrysosporium* revealed the noticeable elongated, highly branched, and ramified hyphae. It is typical for higher filamentous and sporulating fungi, wherein compartments are multinucleated and the fungi grow by extension of their apical compartments (**Figure** **5**). Long and ramified hyphae generally tend to increase the surface area of the fungus, thereby possibly enhancing the interaction with metals.^26^, ^27^ Moreover, substantial deformation of fungal cells could be observed by the presence of shrunken and distorted cell walls in the presence of Cd and depressions in the presence of As and Cr.^28^, ^29^ The binding of the heavy metals to the surface of the cell wall was due to the presence of electronegative functional groups, i.e., carboxyl, hydroxyl, and phosphoryl as evident from FTIR analysis.^32^

**Figure 6:**
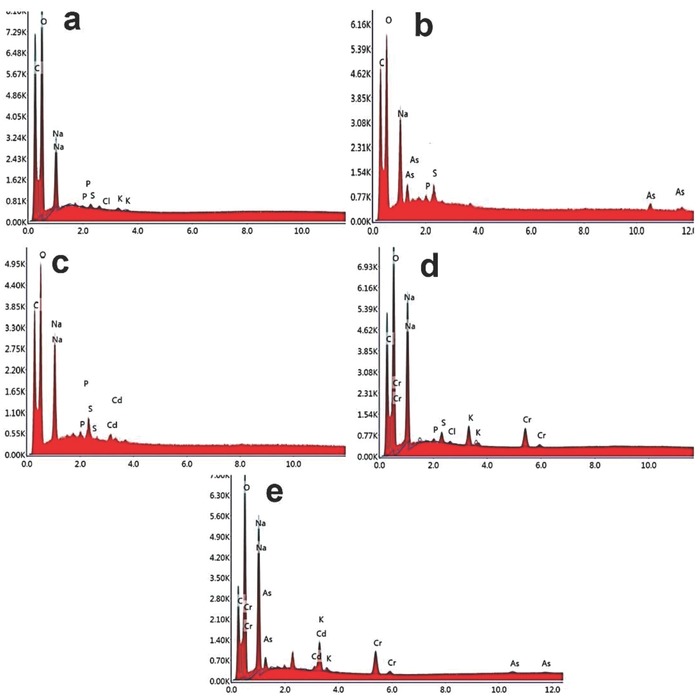
EDXA spectrum of a) control fungal hyphae, b) As‐absorbed hyphae, c) Cd‐absorbed hyphae , d) Cr‐absorbed hyphae, and e) mixed‐metal‐absorbed hyphae.

EDAX spectra of the fungal biomass of *P. chrysosporium* in the presence of three different heavy metals namely As, Cd, and Cr showed the presence of corresponding metal peaks (**Figure** **6**), demonstrating their biosorption on the surface of the biomass. In case of control fungal hyphae, presence of C, O, Na, K, P, and S on the surface of fungal hyphae, revealing the absence of heavy metals in the sample. The presence of As is detected at 1.6, 1.7, 10.5, and 11.8 kV on the surface of fungal hyphae. Cd is detected in EDXA spectrum at 3.2 kV, while Cr is detected at 5.7 and 6.0 kV.

### 2.5 FTIR Analysis

**Figure 7:**
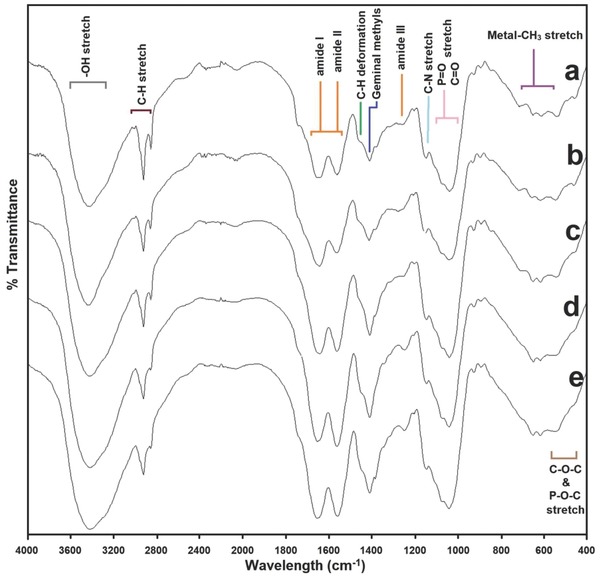
FTIR spectrum of a) control fungal hyphae, b) As‐absorbed hyphae, c) Cd‐absorbed hyphae, d) Cr‐absorbed hyphae, and e) mixed‐metal‐absorbed hyphae.

FTIR spectra of control *P. chrysosporium* (without) and metal absorbed (As, Cd, and Cr) during biosorption are presented in **Figure** **7** in the range of 400–4000 cm^−1^. The intense and broad absorption band at 3426 cm^−1^ in the control sample was due to —OH stretching vibration of the carboxyl group, which is marginally shifted in the metal absorbed biomass spectra.^30^ The peaks at 2925 and 2854 cm^−1^ are assigned for asymmetric and symmetric vibrations of —CH_3_ groups presented in fungal proteins are assigned in control fungal biomass.^31^, ^32^ As shown in Figure 7, these peaks are highly diverged in case of mixed metal biomass sample compared to individual metal biomass samples. Cd, Cr, and mixed metal absorbed biomass marginally shifted for amide I (1644 cm^−1^), amide II (1564 cm^−1^), and amide III (1247 cm^−1^) presented in proteins, revealing the proteins of these samples highly interacted with metals. In fungal cell walls, chitin and its associated polysaccharides contain carbonyl groups, which are also assigned at 1644 cm^−1^.^33^

The peaks at 1381, 1150, and 1042 cm^−1^ are due to —CH_3_ wagging (umbrella deformation), symmetric SO_3_ stretching and C—OH stretching vibrations.^32^ Intensity of P=O (phospholipids) and C=O stretching (polysaccharides) is increased in Cd, Cr, and mixed metal biomass samples as compared to As and control samples. Data suggested that phospholipids and carbohydrates of fungal cells might play important role in biosorption of the metals. Similar results were obtained for germinal methyl that is assigned at 1403 cm^−1^.^33^ 600–800 cm^−1^ region corresponded to the metal‐CH_3_ binding, which mostly showed the interactions between metals and biological components, i.e., proteins, fatty acids, and/or carbohydrates. The peaks at 617 and 542 cm^−1^ are due to O—C—O scissoring and C—O bending vibrations, wherein higher intensity was observed in control and As absorbed samples.^34^, ^35^

Taken together, these findings indicate that surface functional groups (e.g., carboxyl, phosphoryl, and hydroxyl) present on the surface of the *P. chrysosporium* fungal cells play a major role in the bioaccumulation of metals, however with different degree of interaction. Cd‐, Cr‐, and mixed‐metal‐absorbed biomass showed higher degree of interactions with proteins, fatty acids, and carbohydrates. Presumably, As could be metabolized by the fungal cells as the shift of the proteins, fatty acids, and carbohydrates are similar to the control samples. These results are in agreement of our previous results, showing the potential of *P. chrysosporium* to remove As completely from the aqueous solution.

## 3 Conclusion

“*P. chrysosporium*,” is an efficient, biological, and nonhazardous absorbent, which is frequently used for environmental engineering purposes and now, it is introduced to remove toxic metals (As, Cd, and Cr) simultaneously from the aqueous solutions. Response surface methodology provides the efficient optimization of different parameters for the removal of As, Cd, and Cr metal ions either by individually or by simultaneously. Metal absorbed fungal hyphae showed the different morphology as compared to control fungal hyphae. Proteins, carbohydrates, and fatty acids of fungal cells are mostly interacted with Cd, Cr, and mixed metals; however, As might be metabolized by the fungal cells as control and As absorbed samples have similar FTIR profile. The biosorbent used in the present study can efficiently use for the removal of different heavy metals in waste water treatment plant.

## 4 Experimental Section

*Chemicals*: Cd(NO_3_)_2_, NaAsO_2_, K_2_Cr_2_O_7_, Al_2_O_3_, CoCl_2_, CuSO_4_, Ni(NO_3_)_2_, Pb(NO_3_)_2_, SeO_2_, and ZnSO_4_ were procured from Hi‐media Labs (Mumbai, India) and were used as received without further purification. Malt extract, Sabouraud dextrose broth, and other media components were also purchased from the same supplier. Stock solution of individual metals was prepared in autoclaved distilled water and directly used for biosorption experiment.

*Fungal Culture: P. chrysosporium* was obtained as a gift culture from the Institute of Frostbotanik, Gottingen, Germany. The culture was grown on malt extract agar at 30 °C for 8 d. For biomass production, spore suspension of *P. chrysosporium* (2.5 × 10^6^ spores mL^−1^) were inoculated in 100 mL of Sabouraud dextrose medium and incubated at 30 °C and 120 rpm for 10 d. Afterward, obtained fungal pellets (free cells) were repeatedly washed three times with Na‐acetate buffer (pH 5, 50 × 10^−3^ m), dried at 40 °C for 12 h to remove the excessive moisture and further it is used for the biosorption experiments.

*Biosorption Experiments*: To understand the metal binding efficacy using free cells of *P. chrysosporium*, heavy metals, i.e., As, Cd, Cr, Pb, Se, Cu, Al, Co, Ni, and Zn were used for biosorption experiment. In batch experiments, *P. chrysosporium* (300 mg) was incubated with 50 mg L^−1^ of metal in Na‐acetate buffer (pH 5, 50 × 10^−3^ m) at 30 °C and 120 rpm for 1 h. % Biosorption of respective metals were calculated using following formula

(1) $$\;\mathrm{Biosorption}\;\;\left(\%\right)=\;\frac{\left(M_{i}-M_{f}\right)}{M_{i}}\;\times \;100$$

(2) $$\mathrm{Metal}\;\mathrm{uptake}\;\mathrm{efficiency}\;\left(q\right)=\;\frac{\left(M_{i}-M_{f}\right)V_{s}}{W_{b}}$$

where, *M*_i_ = initial metal concentration, *M*_f_ = final metal concentration, *V*_s_ = volume of solution, and *W*_b_ = weight of fungal biomass. Data presented were the mean values from three independent experiments. Standard deviation and error bars were indicated wherever necessary.

*Optimization and Validation Using RSM‐CCD*: From the preliminary results, it was clear that the fungi had higher efficacy to remove As, Cd, and Cr metal ions. Experimental parameters, such as pH of medium, fungal biomass, and metal concentration were optimized using RSM‐CCD, wherein RSM was referred as an optimization process and CCD was mostly used and important design. In RSM‐CCD optimization, specific response was used for the modeling and analysis by changing the concentration of variables and subsequently, the behavior of system analyzed statistically and optimized the variables.^16^ The following equation demonstrated the behavior of the system:

(3) Y=βo+∑βixi+∑βijxixj+∑βiixi2

where, *Y* = predicted response, βo = constant, *βi* = linear coefficient, *βii* = squared coefficient, *βij* = cross product coefficient, *xi* = dimensionless coded value of (*xi*). The above equation was solved using the software Design‐Expert v10.0.5 (State ease Inc., USA). A total of 20 trials were employed to optimize the individual and simultaneous removal of As, Cd, and Cr (**Table** **1**), wherein the range of the independent variables is shown in Table S1 in the Supporting Information. After multivariate optimization, validation of the optimization was conducted by performing the time course study of individual and simultaneous metal removal under respective optimized conditions. Samples were preceded for the metal analysis after biosorption experiments.

**Table 1:** CCD matrix of three independent variables for the individual and simultaneous removal of metals (As, Cd, and Cr) by *P. chrysosporium*

Run no.	pH	Fungal biomass [mg]	Metal concentration [mg L^−1^]	
			Individual	Mixed metala)
1	2.29	300	60	180
2	4	450	30	90
3	4	150	90	270
4	4	150	30	90
5	4	450	90	270
6	6.5	300	110.45	331.36
7	6.5	300	60	180
8	6.5	300	60	180
9	6.5	300	60	180
10	6.5	47.73	60	180
11	6.5	300	60	180
12	6.5	552.26	60	180
13	6.5	300	60	180
14	6.5	300	60	180
15	6.5	300	9.54	28.63
16	9	450	30	90
17	9	450	90	270
18	9	150	30	90
19	9	150	90	270
20	10.70	300	60	180

^a)^ In case mixed metal solution, total of three metal concentrations (mg L^−1^) is displayed in each run.

*Metal Analysis*: After completion of each batch of biosorption experiment, remaining buffer was extracted and centrifuged at 4 °C (8000 rpm, 15 min). Clear supernatant of individual sample was digested using microwave digestion system (Titan MPS 8, Perkin Elmer) and analyzed using inductively coupled plasma spectrophotometer (Optima‐3300 RL, Perkin Elmer) for determination of the metal ion concentration.

*Biosorbent Characterization*: The surface morphology and size of *P. chrysosporium* was observed using field emission gun SEM under vacuum (5 × 10^−3^ Pa) at 10.0 kV with 25 000 × magnification. Untreated and metal‐absorbed fungal hyphae were mounted on a stainless steel stab with a double‐stick tape followed by coating with a thin layer of gold under vacuum to increase the electron conduction and to improve the quality of the microscopic images. Traces of metals on the fungal surface were detected in EDXA (Nano Nova SEM 450, FEI Ltd). The interaction of metals and fungal hyphae was analyzed using FTIR spectroscopy (Spectrum GX, Perkin Elmer). Dried powder of metal absorbed hyphae was mixed with KBr crystals (150 mg) and compressed to form pellet which was further analyzed in midinfrared light region of 400–4000 cm^−1^.

## Conflict of Interest

The authors declare no conflict of interest.

## Supporting information

Supplementary
