# Supplementary material for: Biosorption Potential of Phanerochaete chrysosporium for Arsenic, Cadmium, and Chromium Removal from Aqueous Solutions
Source: Glob Chall. 2018 Oct 25;2(12):1800064. doi: 10.1002/gch2.201800064 (PMC6607372; doi:10.1002/gch2.201800064)
Supplement: Supplementary file 1 — Supplementary [file GCH2-2-1800064-s001.pdf]

Copyright WILEY-VCH Verlag GmbH & Co. KGaA, 69469 Weinheim, Germany, 2018.

Global Challenges
Open Access

Supporting Information

for *Global Challenges,* DOI: 10.1002/gch2.201800064

Biosorption Potential of *Phanerochaete chrysosporium* for Arsenic, Cadmium, and Chromium Removal from Aqueous Solutions

*Darshan M. Rudakiya, Vignesh Iyer, Darsh Shah, Akshaya Gupte, and Kaushik Nath**

Biosorption potential of *Phanerochaete chrysosporium* for arsenic, cadmium and chromium removal from aqueous solutions

Darshan M. Rudakiya [1, ‡], Vignesh Iyer [2, ‡], Darsh Shah [2], Akshaya Gupte [1], Kaushik Nath [2, *]

[‡] contributed equally to this work and are co-first authors.

[1] Department of Microbiology, N. V. Patel College of Pure & Applied Sciences, Vallabh Vidyanagar – 388 120, Anand, Gujarat, India

[2] Department of Chemical Engineering, G. H. Patel College of Engineering & Technology, Vallabh Vidyanagar – 388 120, Anand, Gujarat, India

*Corresponding author

Prof. Kaushik Nath,

Head,

Department of Chemical Engineering,

G. H. Patel College of Engineering & Technology,

Vallabh Vidyanagar – 388 120,

Anand, Gujarat, India

Email: kaushiknath@gcet.ac.in

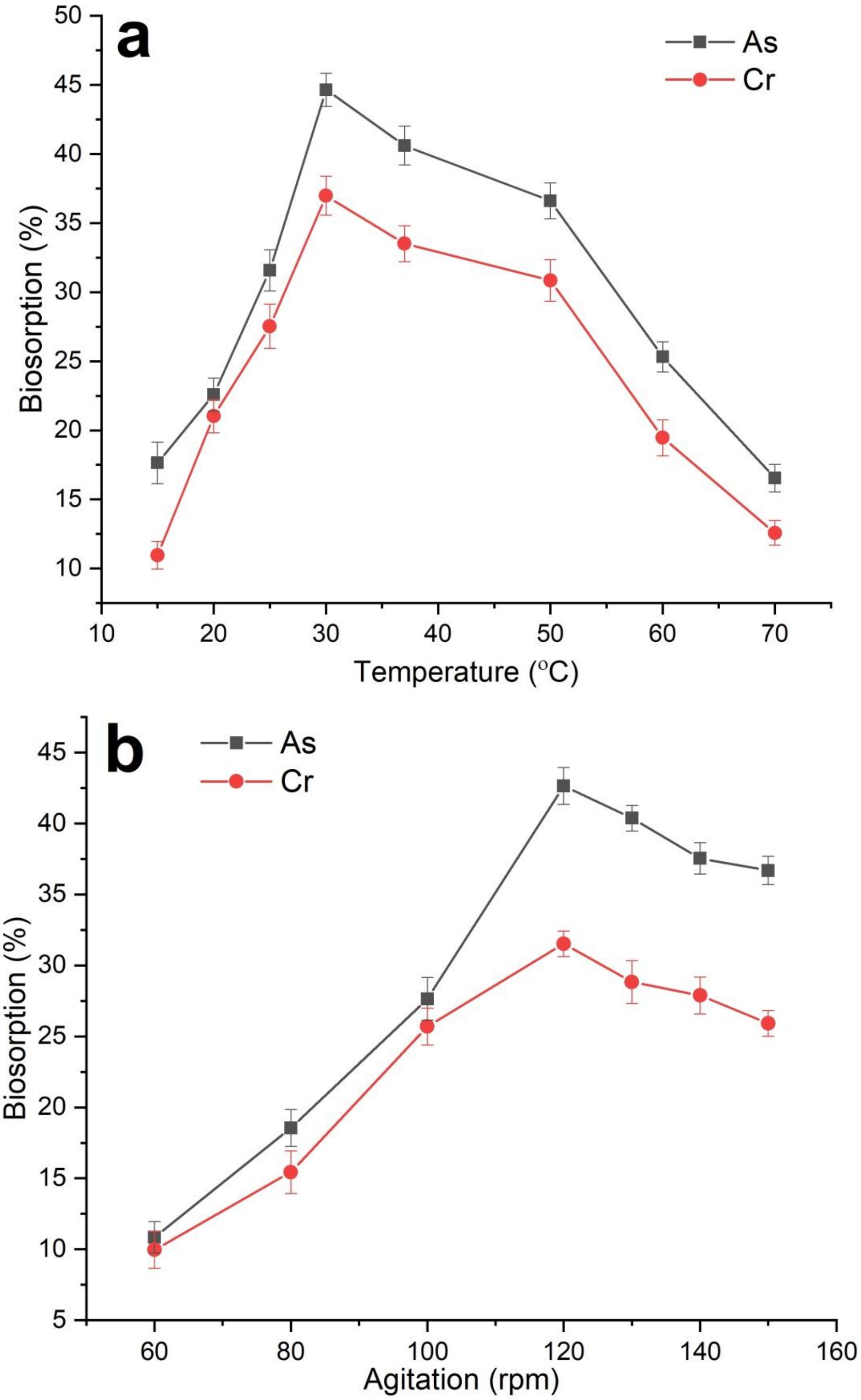

Figure S1 Effect of temperature and agitation on the As and Cr biosorption efficiency by *P. chrysosporium.*

Table S1 Independent components and their values used for individual and simultaneous removal of metal ions in central composite design (CCD).

**Factor Name**	**Units**	**+α**	**+1**	**0**	**-1**	**-α**
pH	-	10.70	9.00	6.50	4.00	2.30
Fungal biomass	mg	552.27	450.00	300.00	150.00	47.73
Metal Concentration (**for individual metal**)	mg L^{-1}	110.45	90.00	60.00	30.00	9.55
Metal Concentration (**for mixed metal**)	mg L^{-1}	331.36	270.00	180.00	90.00	28.64

Table S2 Analysis of variance (ANOVA) for the experiments of CCD for individual metal removal a) As b) Cd and c) Cr

Individual metal removal

(i) As

Source	Sum of Squares	df	Mean Square	F-value	p-value	
**Model**	17846.69	9	1982.97	175.17	< 0.0001	Significant
A-pH	235.01	1	235.01	20.76	0.0010	
B-As Conc	2345.74	1	2345.74	207.22	< 0.0001	
C-Fungal biomass	3346.97	1	3346.97	295.66	< 0.0001	
AB	74.87	1	74.87	6.61	0.0278	
AC	97.78	1	97.78	8.64	0.0148	
BC	105.86	1	105.86	9.35	0.0121	
A^2	11345.47	1	11345.47	1002.22	< 0.0001	
B^2	428.10	1	428.10	37.82	0.0001	
C^2	635.51	1	635.51	56.14	< 0.0001	
**Residual**	113.20	10	11.32			
Lack of Fit	70.74	5	14.15	1.67	0.2946	not significant
Pure Error	42.47	5	8.49			
**Cor Total**	17959.89	19				

**R^2**	0.9937
**Adjusted R^2**	0.9880
**Predicted R^2**	0.9666

(ii) Cd

Source	Sum of Squares	df	Mean Square	F-value	p-value	
**Model**	7010.00	9	778.89	38.92	< 0.0001	Significant
A-pH	1139.86	1	1139.86	56.96	< 0.0001	
B-Cd Conc	1885.71	1	1885.71	94.23	< 0.0001	
C-Fungal biomass	202.77	1	202.77	10.13	0.0098	
AB	43.83	1	43.83	2.19	0.1697	
AC	9.73	1	9.73	0.4862	0.5015	
BC	1.56	1	1.56	0.0778	0.7860	
A^2	3398.62	1	3398.62	169.82	< 0.0001	
B^2	132.61	1	132.61	6.63	0.0277	
C^2	543.85	1	543.85	27.18	0.0004	
**Residual**	200.13	10	20.01			
Lack of Fit	122.52	5	24.50	1.58	0.3143	not significant
Pure Error	77.61	5	15.52			
**Cor Total**	7210.13	19				

**R^2**	0.9722
**Adjusted R^2**	0.9473
**Predicted R^2**	0.8496

(iii) Cr

Source	Sum of Squares	df	Mean Square	F-value	p-value	
**Model**	14255.27	9	1583.92	50.60	< 0.0001	Significant
A-pH	7868.19	1	7868.19	251.36	< 0.0001	
B-Cr Conc	740.02	1	740.02	23.64	0.0007	
C-Fungal biomass	2005.69	1	2005.69	64.07	< 0.0001	
AB	2.74	1	2.74	0.0874	0.7735	
AC	47.18	1	47.18	1.51	0.2477	
BC	171.10	1	171.10	5.47	0.0415	
A^2	2754.51	1	2754.51	88.00	< 0.0001	
B^2	10.04	1	10.04	0.3206	0.5837	
C^2	819.46	1	819.46	26.18	0.0005	
**Residual**	313.03	10	31.30			
Lack of Fit	206.34	5	41.27	1.93	0.2433	not significant
Pure Error	106.69	5	21.34			
**Cor Total**	14568.30	19				

**R^2**	0.9785
**Adjusted R^2**	0.9592
**Predicted R^2**	0.8825

Table S3 Analysis of variance (ANOVA) for the experiments of CCD for mixed metal removal a) As b) Cd and c) Cr

Mixed metal solution:

a) As

Source	Sum of Squares	df	Mean Square	F-value	p-value	
**Model**	3966.54	9	440.73	171.35	< 0.0001	Significant
A-pH	66.56	1	66.56	25.88	0.0005	
B-metal Conc	681.11	1	681.11	264.80	< 0.0001	
C-Fungal biomass	546.04	1	546.04	212.29	< 0.0001	
AB	1.26	1	1.26	0.4884	0.5006	
AC	19.92	1	19.92	7.75	0.0193	
BC	38.00	1	38.00	14.77	0.0032	
A^2	2418.53	1	2418.53	940.29	< 0.0001	
B^2	244.50	1	244.50	95.06	< 0.0001	
C^2	213.91	1	213.91	83.17	< 0.0001	
**Residual**	25.72	10	2.57			
Lack of Fit	4.01	5	0.8028	0.1849	0.9562	not significant
Pure Error	21.71	5	4.34			
**Cor Total**	3992.26	19				

**R^2**	0.9936
**Adjusted R^2**	0.9878
**Predicted R^2**	0.9840

b) Cd

Source	Sum of Squares	df	Mean Square	F-value	p-value	
**Model**	497.14	9	55.24	26.82	< 0.0001	Significant
A-pH	41.95	1	41.95	20.37	0.0011	
B-metal Conc	67.93	1	67.93	32.98	0.0002	
C-Fungal biomass	10.58	1	10.58	5.14	0.0469	
AB	2.90	1	2.90	1.41	0.2630	
AC	0.0109	1	0.0109	0.0053	0.9435	
BC	0.0257	1	0.0257	0.0125	0.9134	
A^2	322.66	1	322.66	156.66	< 0.0001	
B^2	30.61	1	30.61	14.86	0.0032	
C^2	66.28	1	66.28	32.18	0.0002	
**Residual**	20.60	10	2.06			
Lack of Fit	14.02	5	2.80	2.13	0.2128	not significant
Pure Error	6.58	5	1.32			
**Cor Total**	517.73	19				

**R^2**	0.9602
**Adjusted R^2**	0.9244
**Predicted R^2**	0.7757

c) Cr

Source	Sum of Squares	df	Mean Square	F-value	p-value	
**Model**	2129.64	9	236.63	33.77	< 0.0001	Significant
A-pH	225.39	1	225.39	32.17	0.0002	
B-metal Conc	303.94	1	303.94	43.37	< 0.0001	
C-Fungal biomass	350.54	1	350.54	50.03	< 0.0001	
AB	49.45	1	49.45	7.06	0.0240	
AC	55.39	1	55.39	7.90	0.0184	
BC	12.18	1	12.18	1.74	0.2168	
A^2	1024.35	1	1024.35	146.18	< 0.0001	
B^2	108.93	1	108.93	15.54	0.0028	
C^2	125.33	1	125.33	17.89	0.0017	
**Residual**	70.07	10	7.01			
Lack of Fit	47.79	5	9.56	2.15	0.2110	not significant
Pure Error	22.28	5	4.46			
**Cor Total**	2199.71	19				

**R^2**	0.9681
**Adjusted R^2**	0.9395
**Predicted R^2**	0.8120

Table S4 Final equation for coded factors of individual and mixed metal removal by *P. chrysosporium*

Individual metal	Coded Equation
**As**	B_{As} = 92.18 -4.14 (A) -13.10 (B) + 15.65 (C) + 3.05 (AB) -3.49 (AC) + 3.63 (BC) - 28.05 (A^2) - 5.45 (B^2) - 6.64 (C^2)
**Cd**	B_{Cd} = 55.13 - 9.13 (A) - 11.75 (B) + 3.85 (C) + 2.34 (AB) - 1.10 (AC) + 0.44 (BC) - 15.35 (A^2) - 3.03 (B^2) - 6.14 (C^2)
**Cr**	B_{Cr} = 76.37 - 24.00 (A) - 7.361 (B) + 12.11 (C) - 0.58 (AB) + 2.42 (AC) + 4.62 (BC) - 13.82 (A^2) + 0.83 (B^2) - 7.54 (C^2)
**Mixed metal**	
**As**	B_{As} = 44.04 - 2.20 (A) - 7.06 (B) + 6.32 (C) + 0.39 (AB) - 1.57 (AC) + 2.17 (BC) - 12.95 (A^2) - 4.11 (B^2) - 3.85 (C^2)
**Cd**	B_{Cd} = 14.36 - 1.75 (A) - 2.23 (B) + 0.88 (C) + 0.60 (AB) - 0.03 (AC) + 0.05 (BC) - 4.73 (A^2) - 1.45 (B^2) - 2.14 (C^2)
**Cr**	B_{Cr} = 29.15 - 4.06 (A) - 4.71 (B) + 5.06 (C) + 2.48 (AB) - 2.63 (AC) + 1.23 (BC) - 8.43 (A^2) - 2.74 (B^2) - 2.94 (C^2)
